# A tight balance of Karyopherin β1 expression is required in cervical cancer cells

**DOI:** 10.1186/s12885-018-5044-8

**Published:** 2018-11-16

**Authors:** Sarah Carden, Pauline van der Watt, Alicia Chi, Aderonke Ajayi-Smith, Katie Hadley, Virna D. Leaner

**Affiliations:** 1 Division of Medical Biochemistry and Structural Biology, Department of Integrative Biomedical Sciences, Institute of Infectious Disease and Molecular Medicine, SAMRC/UCT Gynaecological Cancer Research Centre, Faculty of Health Sciences, University of Cape Town, Cape Town, South Africa; 2 Division of Medical Biochemistry and Structural Biology, Faculty of Health Sciences, University of Cape Town, Observatory, Cape Town, 7925 South Africa

**Keywords:** Karypherin β1, Overexpression, Cancer biology

## Abstract

**Background:**

Karyopherin β1 (Kpnβ1) is the main nuclear import protein involved in the transport of cargoes from the cytoplasm into the cell nucleus. Previous research has found Kpnβ1 to be significantly overexpressed in cervical cancer and other cancer tissues, and further studies showed that inhibition of Kpnβ1 expression by siRNA resulted in cancer cell death, while non-cancer cells were minimally affected. These results suggest that Kpnβ1 has potential as an anticancer therapeutic target, thus warranting further research into the association between Kpnβ1 expression and cancer progression. Here, the biological effects associated with Kpnβ1 overexpression were investigated in order to further elucidate the relationship between Kpnβ1 and the cancer phenotype.

**Methods:**

To evaluate the effect of Kpnβ1 overexpression on cell biology, cell proliferation, cell cycle, cell morphology and cell adhesion assays were performed. To determine whether Kpnβ1 overexpression influences cell sensitivity to chemotherapeutic agents like Cisplatin, cell viability assays were performed. Expression levels of key proteins were analysed by Western blot analysis.

**Results:**

Our data revealed that Kpnβ1 overexpression, above that which was already detected in cancer cells, resulted in reduced proliferation of cervical cancer cells. Likewise, normal epithelial cells showed reduced proliferation after Kpnβ1 overxpression. Reduced cancer cell proliferation was associated with a delay in cell cycle progression, as well as changes in the morphology and adhesion properties of cells. Additionally, Kpnβ1 overexpressing HeLa cells exhibited increased sensitivity to cisplatin, as shown by decreased cell viability and increased apoptosis, where p53 and p21 inhibition reduced and enhanced cell sensitivity to Cisplatin, respectively.

**Conclusions:**

Overall, our results suggest that a tight balance of Kpnβ1 expression is required for cellular function, and that perturbation of this balance results in negative effects associated with a variety of biological processes.

**Electronic supplementary material:**

The online version of this article (10.1186/s12885-018-5044-8) contains supplementary material, which is available to authorized users.

## Background

The Karyopherin protein superfamily consists of a large number of soluble transport factors that shuttle proteins and certain RNAs through the nuclear pore complex (NPC). Karyopherin β1 (Kpnβ1), the primary nuclear import protein in interphase cells, imports cargoes that contain a nuclear localisation sequence (NLS) across the nuclear membrane and into the nucleus [1]. In the classical nuclear import pathway, recognition of an NLS, which most commonly consists of a number of clusters of basic amino acids and is termed the classical NLS (cNLS), by a Karyopherin α (Kpnα) isoform is required for Kpnβ1-mediated nuclear import [2, 3]. After Kpnα binds to the NLS-containing cargo, forming a bridge between the cargo and Kpnβ1, Kpnβ1 interacts with nucleoporins (Nups) on the NPC to allow for transfer of the cargo through the channel. Once in the nucleus, binding of RanGTP results in dissociation of the trimeric complex and release of the cargo [4]. Kpnβ1 and Kpnα are then recycled back to the cytoplasm for another round of nuclear import. Alternatively to classical nuclear import, some cargoes are able to bind Kpnβ1 directly (independent of Kpnα; non-classical pathway), or utilise other members of the Karyopherin β family for their nuclear import.

Studies have shown that in addition to its role as a nuclear importer, Kpnβ1 has an equally vital set of roles during mitosis, acting as a negative regulator for various mitotic processes from spindle assembly and regulation to nuclear membrane and nuclear pore reformation, where in most cases it is counter-regulated by the small regulatory GTPase, Ran [5, 6]. Previous studies have shown that disruption of Kpnβ1 expression (by loss of endogenous Kpnβ1 through siRNA-mediated knockdown, or transient overexpression of Kpnβ1 using plasmid-based transfection), resulted in the appearance of distinct mitotic defects, such as multipolar spindles, chromosome misalignment, and lagging or mis-segregation of chromosomes in anaphase/telophase [7–9]. These results suggest that a balance of Kpnβ1 expression may be necessary for correct cell functioning.

It has been shown that Kpnβ1 expression is elevated in cervical cancer tissue, as well as a number of transformed and cancer cell lines, suggesting its association with the cancer phenotype [10–12] . We previously showed that inhibition of Kpnβ1 protein expression leads to cancer cell death via apoptosis [10], and Kuusisto and Jans (2015) showed that faster growing tumour cells expressing high levels of Kpnβ1 are more dependent on, or “addicted to”, Kpnβ1 than their non-transformed counterparts [13]. These results demonstrate that increased expression, and thus activity, of Kpnβ1 likely plays a role in cancer cell proliferation, making Kpnβ1 an attractive anticancer therapeutic target. Interestingly, siRNA-mediated knockdown of Kpnβ1 leaves normal cells relatively unaffected [9, 10]. It is possible that cancer cells have devised a mechanism to cope with their increased metabolic and proliferative demands by enhancing expression of various proteins, among them Kpnβ1, potentially explaining why depletion of Kpnβ1 has such negative effects. In contrast, it appears as if normal cells are less reliant on Kpnβ1 for processes such as proliferation.

While a number of studies have revealed the importance of Kpnβ1 in cancer cell survival and proliferation, and described its potential as an anticancer target, the precise role that Kpnβ1 plays in cellular transformation and cancer progression is largely unclear. In this study, we investigated the biological effects associated with Kpnβ1 overexpression in order to further elucidate the relationship between Kpnβ1 and cancer progression. While it is evident from previous work that cancer cells require Kpnβ1 for their proliferation and survival, it is unclear whether overexpression of Kpnβ1 provides a growth advantage to cancer cells, or leads to phenotypes associated with cellular transformation. In this study, we show that Kpnβ1 overexpression in fact leads to negative effects associated with the functioning of cervical cancer cells, and thus a precise balance of Kpnβ1 expression and activity appears to be essential for the correct functioning of these cells. Disruption of this balance in either direction has detrimental effects on many cellular processes.

## Methods

### Cell lines and cell culture

The human cervical carcinoma cell lines, HeLa (ATCC CCL-2) and CaSki (ATCC CRL-1550), and normal primary (ARPE19) (ATCC CRL-2302) and immortalised (hTERT-RPE-1) (ATCC CRL-4000) epithelial cells were obtained from the American Type Culture Collection (ATCC). Cancer cells were cultured in Dulbecco’s Modified Eagle’s Medium (DMEM), supplemented with penicillin (100 U/ml), streptomycin (100 μg/ml) and 10% Fetal Calf Serum (FCS) (HiClone, Thermo Scientific, USA). Normal cells were cultured in DMEM/F12 media, supplemented with penicillin, streptomycin and 10% FCS. hTERT-RPE-1 cells were maintained in the presence of 10 μg/ml Hygromycin B. Cells were incubated at 37 °C in 5% CO_2_. Cancer cell lines were authenticated by DNA profiling using the Cell ID system (Promega, USA).

### Generation of stable Kpnβ1 overexpressing cell lines

The plasmid used for overexpression of Kpnβ1 (pEFIRES-Kpnβ1-EGFP) was generated by inserting *Sac*I- and *Not*I-digested human Kpnβ1-EGFP, released from the plasmid pEGFP-Kpnβ1 (kind gift from Patrizia Lavia, Institute of Molecular Biology and Pathology, National Research Council of Italy, Rome, Italy) [7], into the pEFIRES plasmid (kind gift from Yosef Shaul, Weizmann Institute of Science, Israel) [14]. The pEFIRES plasmid was used in order to allow for both the Kpnβ1-EGFP fusion gene and the puromycin resistance gene to be transcribed as a single mRNA transcript. The presence of an internal ribosome entry site (IRES) upstream of the puromycin resistance gene ensures that puromycin-resistant clones express high levels of the recombinant protein, Kpnβ1-EGFP. pEFIRES-EGFP was used as a control. For the establishment of cells stably expressing Kpnβ1-EGFP, cells were transfected with the pEFIRES-EGFP and pEFIRES-Kpnβ1-EGFP constructs using Genecellin Transfection Reagent (Celtic Molecular Diagnostics) or TransFectin Lipid Reagent (Bio-Rad), and thereafter positively transfected cells were selected with Puromycin (Calbiochem, Merck). Pools of stably transfected cells were maintained in 0.5 μg/ml Puromycin.

### Fluorescence microscopy

To analyse EGFP-tagged protein localisation using fluorescent microscopy, cells were grown on glass coverslips and fixed in 4% paraformaldehyde. Coverslips were mounted onto glass slides using Mowiol. Images were captured using the AxioVision 4.7 software (Zeiss, Oberkochen, Germany) under 100 x oil immersion.

### Western blot analysis

Western Blot analyses were performed using rabbit anti-Kpnβ1 (H300) (Santa Cruz Biotechnology, sc-11367), mouse anti-GAPDH (0411) (Santa Cruz Biotechnology, sc-47724), rabbit anti-GFP (FL) (Santa Cruz Biotechnology, sc-8334), rabbit anti-Cyclin A (H-432) (sc-751), rabbit anti-Cyclin D1 (HD11) (sc-246), rabbit anti-pHisH3 (Ser-10)-R (Santa Cruz Biotechnology, sc-8656), rabbit anti-Mcl-1 (H-260) (Santa Cruz Biotechnology, sc-20679), rabbit anti-E-cadherin (H-108) (Santa Cruz Biotechnology, sc-7870), mouse anti-Vimentin (V-9) (Santa Cruz Biotechnology, sc-6260), rabbit anti-PARP1/2 (H-250) (Santa Cruz Biotechnology, sc-7150), mouse anti-p53 (DakoCytomation, M7001), rabbit anti-p21 (H-164) (Santa Cruz Biotechnology, sc-756), rabbit anti-γH2AX (Ser-139) (Cell Signaling, 2577S), and rabbit anti-TFIID (TBP) (N-12) (Santa Cruz Biotechnology, sc-204) antibodies.

### Luciferase assays

To assay for NFAT luciferase activity, cells were transfected with 50 ng GFP-NFAT (Addgene plasmid #24219; gift of Jerry Crabtree [15]), 50 ng NFAT-luciferase (Addgene plasmid #10959; gift of Toren Finkel [16]), and 5 ng pRL-TK (encoding *Renilla* luciferase; Promega, USA). To assay for AP-1, NFκB p65 and p53 luciferase activity, cells were transfected with either 100 ng AP1-luciferase reporter construct (containing four copies of the AP-1 binding site; gift of Michael Birrer [17]), 100 ng NFκB p65 luciferase reporter construct (containing 5 copies of the p65 binding site; Promega, USA) or 100 ng p53-luciferase reporter construct (Addgene plasmid #16442; gift of Bert Vogelstein [18]), as well as 5 ng pRL-TK. For NFκB p65 luciferase assays, cells were stimulated with 0.5 μM PMA (Sigma-Aldrich, USA) for 3 h, at 24 h post transfection. Luciferase activity was assayed using the Dual-Luciferase Reporter assay system (according to the manufacturer’s instructions; Promega, USA). Luciferase readings measured using the Veritas microplate luminometer (Promega, USA) were normalised to *Renilla* luciferase readings.

### Trypan blue cell viability assays

To assay for the number of viable cells, cells were trypsinised and incubated with 0.4% Trypan Blue (Merck Millipore, USA). Viable (white) and non-viable (blue) cells were counted using a haemocytometer, and the number of live cells at various time points was recorded.

### MTT proliferation assays

To allow for anchorage-independent growth, cells were resuspended in 1% methyl cellulose-containing media and were plated onto Poly (2-hydroxyethyl methacrylate) (Poly-HEMA) (Sigma-Aldrich, USA) coated 96-well plates. The number of colonies formed at various time points post plating were measured using the MTT reagent (according to the manufacturer’s instructions; Sigma-Aldrich, USA).

Adherent growth as a result of Kpnβ1 overexpression was determined using the MTT proliferation assay (according to the manufacturer’s instructions; Sigma-Aldrich, USA).

For the analysis of the effect of p53 and p21 inhibition on Cisplatin-induced cell death, cells were either co-treated with Pifithrin α (Sigma) and Cisplatin, or transfected with control or p21 siRNA (Santa Cruz Biotechnology), using Transfectin (BioRad, USA) transfection reagent, and treated with Cisplatin 48 h post-transfection. MTT assays were performed 24 h after Cisplatin treatment.

### Cell cycle analysis

Cells were synchronised with 2 mM Thymidine (Sigma-Aldrich, USA), and released into fresh media. Cells (and floaters) were harvested and fixed in 100% ethanol overnight. Fixed cells were treated with 50 μg/ml RNase and stained with propidium iodide. Cell cycle profiles were analysed using a BD Accuri Flow Cytometer (Beckman Coulter, Fullerton, CA, USA). Quantification of the percentage of cells at different cell cycle stages was performed using the ModFit LT 3.3 software (Verity Software House, USA).

### Phalloidin staining of F-actin

Cells were fixed and washed twice in 0.04% PBST before blocking in 1% BSA for 30 min. Actin was labeled with 50 ng/ml Phalloidin-Tetramethylrodamine B isothiocyanate (Phalloidin) (Sigma-Aldrich, USA) in 1% BSA for 30 min at room temperature. Cell nuclei were stained with 100 ng/ml DAPI and coverslips mounted onto glass slides using Mowiol. Phalloidin images were viewed using the Zeiss Inverted Fluorescence Microscope under 100 x oil immersion and images captured using the AxioVision 4.7 software (Zeiss, Germany).

### Cell adhesion assays

Cells were plated on uncoated plates and allowed to adhere for 1 h at 37 °C. Thereafter, the medium was removed from all wells and ‘washed’ cells were rinsed twice with PBS before fixation of all cells in 0.5 ml fixation solution (acetic acid/methanol (1:7)) for 5 min followed by staining with 0.5% crystal violet solution for 2 h at room temperature. Plates were rinsed in water and left to dry overnight. The number of cells over various fields of view were counted using a light microscope and normalized to the number of ‘unwashed’ cells, in order to control for total cells plated.

### In vitro scratch wound healing assay

Cells were grown to approximately 90% confluence, wounded (at time 0 h) using a pipette tip, and treated with 5 μg/ml Mitomycin C (Sigma). To record scratch wound closure, images were captured at 0, 3, 6 and 24 h time points and gap size measured. Each time point was normalized to the time 0 gap size.

### IC_50_ determination assays

For the determination of drug IC_50_ values, cells were treated with varying concentrations of cisplatin for a period of 48 h, after which the MTT assay was performed (according to the manufacturer’s instructions; Sigma-Aldrich, USA). IC_50_ curves were generated using GraphPad Prism (GraphPad Software Inc., USA).

### Nuclear and cytoplasmic protein fractionation

Cells were grown to 80% confluency, trypsinised, and the cell pellet resuspended in at least 6 volumes harvest buffer (10 mM HEPES, pH 7.9, 50 mM NaCl, 0.5 M Sucrose, 0.1 mM EDTA, 0.5% Triton X-100). Lysates were incubated on ice for 5 min, followed by centrifugation. The supernatant was kept aside as the cytoplasmic fraction, and the pellet was resuspended in 500 μl buffer A (10 mM HEPES, pH 7.9, 10 mM KCl, 0.1 mM EDTA, 0.1 mM EGTA). Centrifugation was performed followed by resuspension of the pellet in 4 volumes buffer C (10 mM HEPES, pH 7.9, 500 mM NaCl, 0.1 mM EDTA, 0.1 mM EGTA, 0.1% NP40). Samples were vortexed for 15 min, followed by centrifugation, whereupon the supernatant was kept as the nuclear extract.

### Statistical analysis

For all data comparisons, the Student’s t test was performed using Microsoft Excel. Data was presented as the mean ± SEM. Unless where otherwise stated, experiments were performed in triplicate or quadruplicate and repeated at least three independent times. A *p* value of < 0.05 was considered statistically significant.

## Results

### Exogenous Kpnβ1 enhances the nuclear import of known Kpnβ1 cargoes in cervical cancer cells

**Fig. 1:**
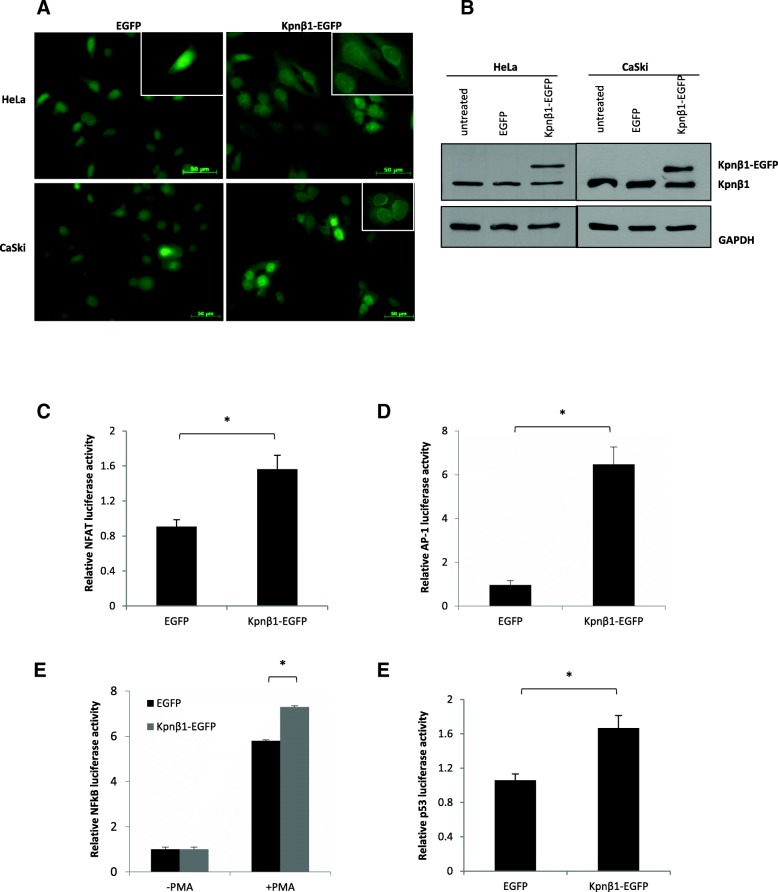
Kpnβ1 localizes to the nucleus and nuclear membrane in cervical cancer cells and enhances the nuclear import of known Kpnβ1 cargoes. **a** Immunofluorescence analysis was used to determine GFP expression across the HeLa and CaSki EGFP and Kpnβ1-EGFP stable cell lines. Representative GFP-Alexa 488 images are shown for each cell line. Enlarged images in the top right-hand corner show perinuclear localization in representative Kpnβ1-EGFP cells. **b** Western blot analysis was used to determine endogenous Kpnβ1 (97 kDa) and exogenous Kpnβ1-EGFP (129.7 kDa) expression levels across the HeLa and CaSki EGFP and Kpnβ1-EGFP cell lines. The first lane represents protein harvested from untransfected cells. GAPDH was used as a control for loading. **c - f** Stable expression of Kpnβ1-EGFP in HeLa cells resulted in a significant increase in NFAT (**c**), AP-1 (**d**), PMA-stimulated NFκB p65 (**e**) and p53 (**f**) promoter-driven luciferase activity. Results shown represent the mean ± SEM (*p < 0.05)

HeLa and CaSki cell lines stably expressing Kpnβ1-EGFP were established to study the effects of Kpnβ1 overexpression on various biological phenotypes. To confirm the presence of EGFP and Kpnβ1-EGFP, cells were visualised using fluorescence microscopy. HeLa and CaSki cells expressing EGFP showed diffuse fluorescence that was both cytoplasmic and nuclear (predominantly nuclear), probably due to the fact that EGFP is small (~ 32 kDa) and can thus passively diffuse across the NPC. Cells expressing Kpnβ1-EGFP showed nuclear and cytoplasmic localization of exogenous Kpnβ1, as well as a distinct perinuclear band (Fig. 1a). This correlates with Kpnβ1’s function as a nuclear transport protein. Western blot analysis revealed that Kpnβ1-EGFP was expressed at approximately equivalent levels to that of endogenous Kpnβ1 in both HeLa and CaSki cells (Fig. 1b).

To investigate whether overexpression of Kpnβ1 leads to enhanced Kpnβ1 functionality (i.e. Kpnβ1-mediated nuclear import), the nuclear activity of various Kpnβ1 cargoes was assayed in HeLa EGFP and Kpnβ1-EGFP cells. Luciferase assays were performed to assay for nuclear activity of Kpnβ1 cargoes NFAT [14], AP-1 [19], NFκB p65 [20] and p53 [21, 22]. HeLa cells stably expressing Kpnβ1-EGFP showed a significant increase in the nuclear activity of NFAT (Fig. 1c), AP-1 (Fig. 1d), PMA-induced NFκB p65 (Fig. 1e) and p53 (Fig. 1f), thereby suggesting that overexpession of Kpnβ1, by expression of exogenous Kpnβ1-EGFP, leads to increased nuclear activity of Kpnβ1 cargo proteins.

### Overexpression of Kpnβ1 results in reduced proliferation of cervical cancer and normal epithelial cells

**Fig. 2:**
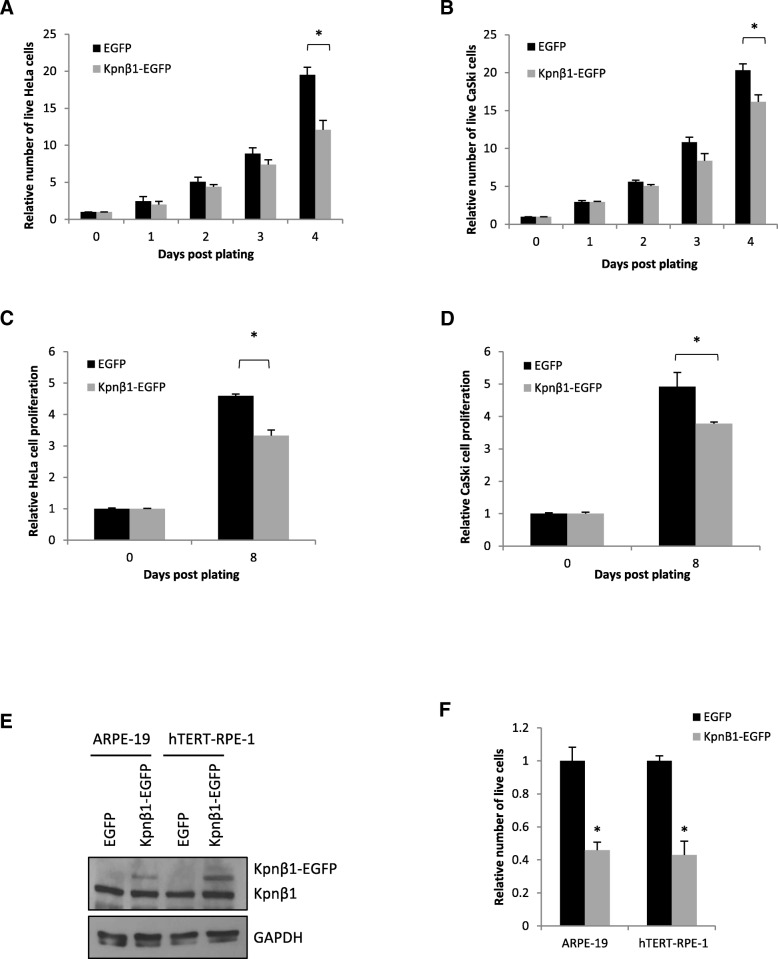
Overexpression of Kpnβ1 results in reduced proliferation of cervical cancer and normal epithelial cells. **a, b** Trypan Blue assays revealed a significant reduction in the number of live HeLa (**a**) and CaSki (**b**) cells expressing Kpnβ1-EGFP compared to EGFP. Cells were counted for a period of up to 4 days post plating. **c, d** MTT cell proliferation assays reveal a significant reduction in the anchorage-independent proliferation of HeLa (**c**) and CaSki (**d**) cells stably overexpressing Kpnβ1. Cells growing adherently were plated in 1% methylcellulose-containing media into 96-well plates coated in 12 mg/ml poly-HEMA. Cell proliferation was assayed by the addition of the MTT reagent for a period of up to 8 days. Results shown represent the mean ± SEM (*p < 0.05). **e** Western blot analysis reveals moderate Kpnβ1 overexpression in ARPE-19 and hTERT-RPE-1 normal epithelial cell lines. **f** Trypan blue assays show a significant reduction in ARPT-19 and hTERT-RPE-1 cell number 96 h after transient Kpnβ1 overexpression. Results shown represent the mean ± SEM (*p < 0.05)

Since high levels of Kpnβ1 are required for cancer cell survival and proliferation [10], it was anticipated that overexpression might confer a further growth advantage. To determine the effect of Kpnβ1 overexpression on cell proliferation, trypan blue cell viability assays were performed. Interestingly, results revealed that overexpression of Kpnβ1 resulted in a significant reduction in the number of adherent HeLa and CaSki cells (Fig. 2a, b). The number of live cells decreased and minimal dead cells were observed, suggesting an inhibition of proliferation rather than induction of cell death. When the effect of Kpnβ1 overexpression on cell proliferation was expanded to look at cells grown under anchorage-independent conditions, a similar result was observed; a significant reduction in proliferation of HeLa and CaSki cells stably expressing Kpnβ1-EGFP (Fig. 2c, d).

Since fast proliferating cancer cells already display high levels of Kpnβ1, it was next determined whether normal epithelial cells transfected with exogenous Kpnβ1 might display increased proliferation. Surprisingly, Kpnβ1 overexpression in the primary and immortalised epithelial cell lines, ARPE-19 and hTERT-RPE-1, respectively, resulted in significantly reduced cell proliferation, similar to that observed in the cancer cell lines, even after only modest overexpression (Fig. 2e, f). These results suggest that Kpnβ1 overexpression alone is not beneficial to cells, and in fact inhibits cell proliferation.

### Overexpression of Kpnβ1 results a delay in cervical cancer cell cycle progression

**Fig. 3:**
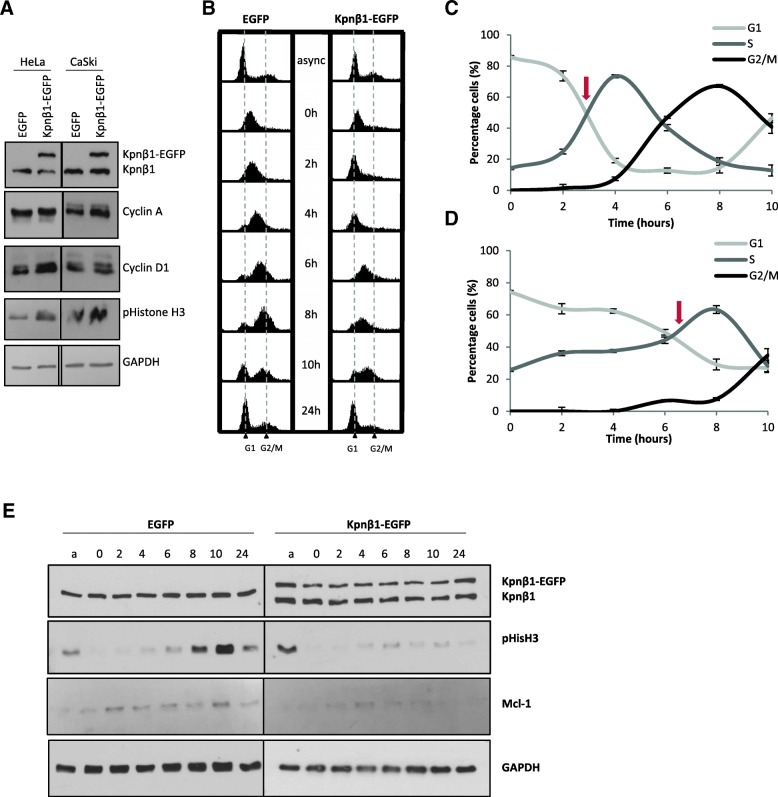
Overexpression of Kpnβ1 results in a delay in cell cycle progression. **a** Protein was harvested from HeLa and CaSki EGFP and Kpnβ1-EGFP cells and cell cycle markers investigated by western blot analysis. Cyclin A, cyclin D1 and pHistoneH3 were all expressed at slightly higher levels in the overexpressing cells compared to the EGFP control cells. GAPDH was used to control for protein loading. **b** Flow cytometric analysis of HeLa EGFP and HeLa Kpnβ1-EGFP cells following synchronization into late G1 and release into S phase. Cells were synchronized with 2 mM thymidine, harvested at various time points post release and examined by FACS analysis. An asynchronous culture of cells (async) was used for comparison. **c** and **d** Line graphs represent quantification of the percentage of cells in each phase of the cell cycle for EGFP (**b**) and Kpnβ1-EGFP (**c**) cells. Arrows represent the approximate point where 50% cells had exited the G1 phase. Results shown represent the mean ± SD. **e** Western blot analysis was used to determine the expression levels of cell cycle associated proteins at various time points post release following thymidine synchronization, in HeLa EGFP and Kpnβ1-EGFP cells. Protein harvested from an asynchronous culture of cells (**a**) is shown for comparison. GAPDH was used as a control for loading

Due to the suppression of cell proliferation upon exogenous Kpnβ1 expression, the effects of Kpnβ1 overexpression on cell cycle progression were next investigated. Using FACS analysis, asynchronous cells revealed only minor changes when Kpnβ1 was overexpressed (data not shown), but despite this, it was possible that the cell cycle was being arrested at several phases after Kpnβ1 overexpression. Various cell cycle markers in these cells were thus investigated by western blot analysis. Levels of Cyclin A, Cyclin D1 and pS10-Histone-H3 (pHisH3), a marker for cells in mitosis, were all slightly elevated in the HeLa and CaSki Kpnβ1-EGFP cells (Fig. 3a), suggesting multiple phases of the cell cycle were affected by Kpnβ1 overexpression. To investigate this further, HeLa EGFP and Kpnβ1-EGFP cells were synchronized to late G1/early S phase using a thymidine block and harvested at various time points post release into complete medium. FACS analysis of synchronized cells revealed that Kpnβ1 overexpression resulted in a prolonged delay or progression from the G1/S block, as Kpnβ1-EGFP expressing cells progressed through the cell cycle much slower than the EGFP expressing cells (Fig. 3b). Quantification of the percentage of HeLa EGFP and Kpnβ1-EGFP cells in each phase of the cell cycle showed that while 50% of EGFP expressing cells had exited G1 phase and entered S phase by approximately 3 h, it took approximately 6 h for the Kpnβ1-EGFP expressing cells to reach the same stage (see arrows; Fig. 3c and d). These results suggest that Kpnβ1-EGFP expressing cells show delayed progression through G1/S and entry into G2/M phase of the cell cycle.

Western blot analysis confirmed that Kpnβ1 overexpression resulted in a delay in cell cycle progression. After release from the thymidine block, pS10-Histone-H3 (pHisH3) peaked at 10 h post release in EGFP cells, but remained low throughout the whole time-course in Kpnβ1-EGFP cells indicating that the cells did not reach M-phase within 10 h post release (Fig 3e). Mcl-1 has been previously shown to increase during S and G2 phase and peak at mitosis [23], and this same trend was observed in EGFP cells (for the Mcl-1 long isoform; the short isoform was not detected). Kpnβ1-EGFP cells, however, showed a decrease in expression from the baseline levels observed in asynchronous cells, suggesting that Mcl-1-L may be downregulated or degraded in response to Kpnβ1 overexpression. Interestingly, degradation of Mcl-1 has been shown to be induced under a number of cell stress conditions [23, 24].

These results support a central role for Kpnβ1 in regulating the proliferation and cell cycle progression of cervical cancer cells, such that when its expression is enhanced, or inhibited [9, 10, 25], cancer cell proliferation and cell cycle progression is reduced.

### Kpnβ1 overexpression affects cell morphology and actin reorganization

**Fig. 4:**
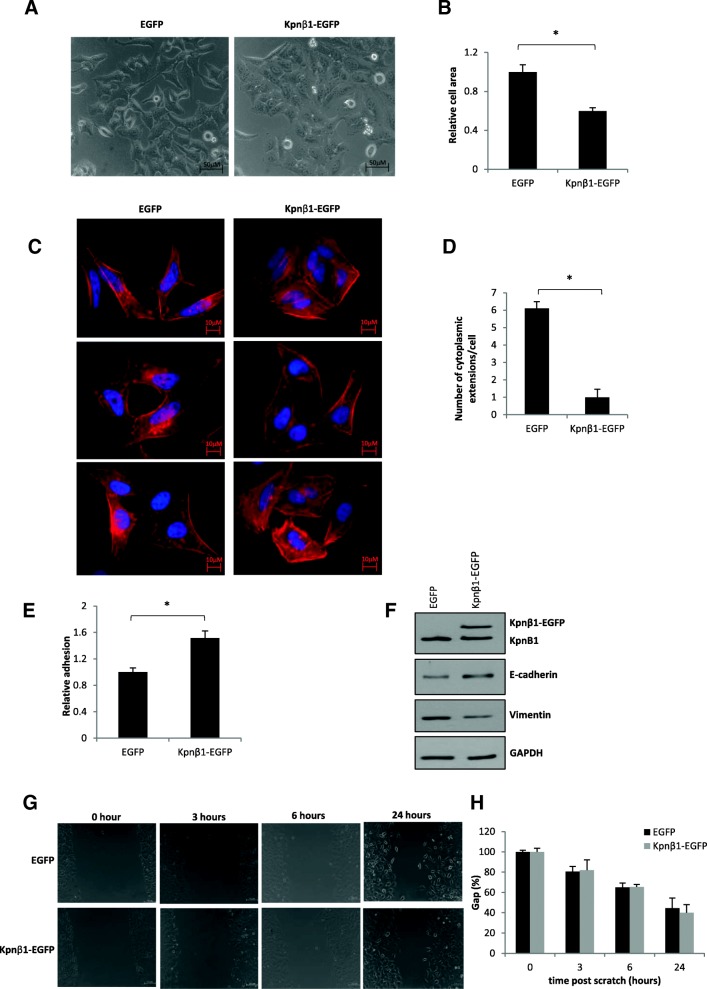
Overexpression of Kpnβ1 results in changes in the morphology and adhesion properties of cervical cancer cells. **a** Phase contrast images showing HeLa EGFP and Kpnβ1-EGFP cells, taken 48 h post plating. Cells were viewed at 20 x magnification using the Zeiss Primovert inverted phase microscope. **b** Quantification of relative HeLa cell area ± SEM of forty cells from each condition was performed using the AxioVision 4.7 software (*p < 0.05). **c** Fluorescent staining of polymeric F-actin using phalloidin (red) in EGFP and Kpnβ1-EGFP expressing HeLa cells. DAPI stain was used to visualize the cell nuclei (blue). **d** Quantification of the number of cytoplasmic protrusions from the captured fluorescent images. Results shown represent the mean ± SEM over fifteen fields of view (*p < 0.05). **e** Relative cell adhesion of HeLa EGFP and Kpnβ1-EGFP cells. Adherent cells (on uncoated tissue culture plates) were fixed (after removing non-adherent cells by washing) and stained with 0.5% crystal violet solution. Cells over ten fields of view, viewed at 10 x magnification, were counted using ImageJ and normalized to unwashed cells. Results shown represent the mean ± SEM (*p < 0.05)**. f** Western blot analysis showing E-cadherin and Vimentin expression in Kpnβ1-overexpressing cells. GAPDH was used as a control for loading. **g** An in vitro scratch wound healing assay was performed and showed no change in migration of HeLa EGFP and Kpnβ1-EGFP cells within a 24 h period. **h** Quantification of the scratch wound healing assay in G

Due to the negative effects of Kpnβ1 overexpression on cancer cell proliferation and cell cycle progression, the effects of Kpnβ1 overexpression on additional biological phenotypes, including cell morphology and actin organization, were next analysed. Phase contrast microscopy of HeLa and CaSki cells stably overexpressing Kpnβ1 showed distinct changes in cell morphology, with cells becoming smaller and more tightly packed (Fig. 4a, Additional file 1: Figure S1A). Quantification of the changes in cell area showed that Kpnβ1 overexpression resulted in a significant decrease in cell area when compared to the area of HeLa and CaSki EGFP cells (Fig. 4b, Additional file 1: Figure S1B).

Changes in cell morphology are often driven by rearrangement of the cytoskeleton; thus immunofluorescence actin staining assays were performed using phalloidin, a high-affinity filamentous actin (F-actin) probe, to investigate actin reorganization in HeLa and CaSki cells stably overexpressing Kpnβ1. Phalloidin staining revealed changes in cell shape, as well as a reduction in the number of actin-rich cytoplasmic extensions seen at the edges of the elongated, spindle-shaped control cells, more evident in HeLa cells (Fig. 4c, Additional file 1: Figure S1C). Quantification of these changes showed that Kpnβ1 overexpression resulted in a significant decrease in cell area (data not shown), as well as a significant decrease in the number of cytoplasmic protrusions (Fig. 4d, Additional file 1: Figure S1D). This data confirms the changes in cell morphology and size seen using phase contrast microscopy, and suggests that Kpnβ1 (or the changes associated with overexpression of Kpnβ1) plays a role in actin reorganisation.

### Overexpression of Kpnβ1 results in changes in cell adhesion properties

The observed morphological changes between EGFP and Kpnβ1-EGFP expressing cells suggests that overexpression of Kpnβ1 causes cells to pack tighter and form clusters, which indicates the possibility of tight junction formation and increased cell adhesion. Thus, to investigate whether the observed morphological changes were associated with any changes in cell adhesion, adhesion assays using uncoated cell culture dishes were used to determine overall changes in the adhesion properties of cells (particularly those involving cell-surface/cell-substrate interactions). Results show that HeLa and CaSki cells overexpressing Kpnβ1 were significantly more adherent compared to EGFP expressing cells (Fig. 4e, Additional file 1: Figure S1E). Furthermore, the expression levels of various proteins known to be involved in adhesion, such as E-cadherin (plays an important role in promoting cell-cell adhesion [26]), and Vimentin (a marker of mesenchymally derived, non-adherent cells [27]), were also investigated. Western blot analysis revealed that E-cadherin levels were elevated in HeLa and CaSki cells overexpressing Kpnβ1, while Vimentin levels were decreased (Fig. 4f, Additional file 1: Figure S1F). To determine whether the altered cell adhesion properties and EMT protein profiles of Kpnβ1-EGFP expressing cells might affect cell migration, a scratch wound healing assay was performed. Interestingly, no change in cell migration was identified in the Kpnβ1-EGFP compared to control EGFP cells (Fig. 4g, h, Additional file 1: Figure S1G, H). These results suggest that dysregulation of Kpnβ1 expression results in altered processes associated with cell-surface (ie. cell to extracellular matrix) and cell-cell adhesion, but does not affect the migration capabilities of cervical cancer cells.

### Kpnβ1 overexpression sensitizes HeLa cells to cisplatin treatment

**Fig. 5:**
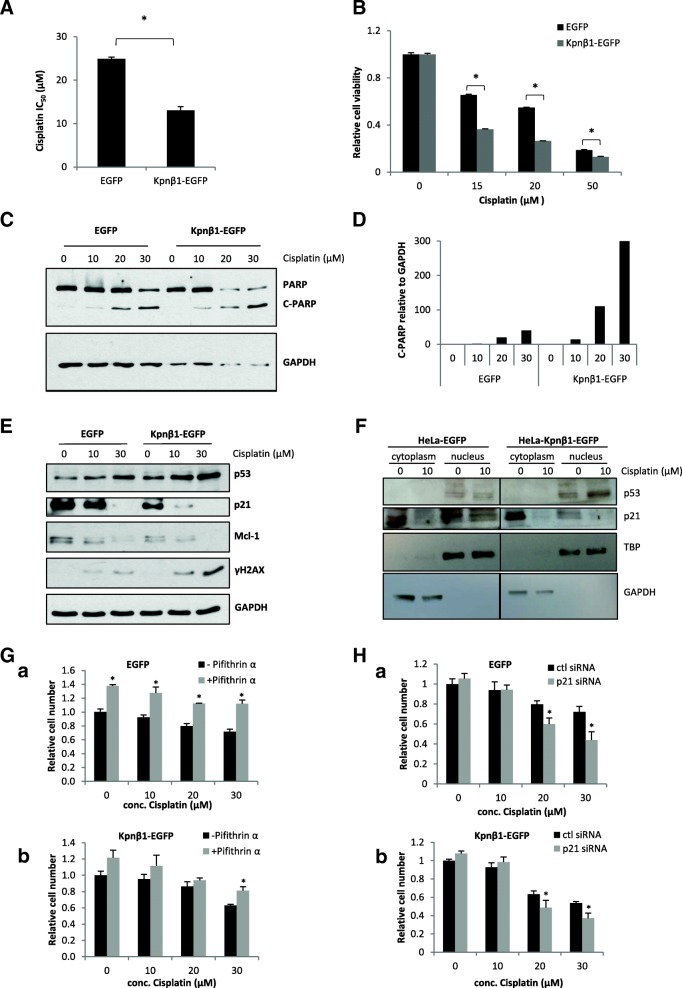
Cisplatin sensitivity in Kpnβ1 overexpressing cells. **A** The IC_50_ value for cisplatin was determined for HeLa EGFP and HeLa Kpnβ1-EGFP cells. **B** Cell viability for EGFP and Kpnβ1-EGFP cells was analyzed 48 h after cisplatin treatment via an MTT assay, and results were normalized to the viability of the untreated cells. Results shown for A and B represent the mean ± SEM (*p < 0.05). **C** PARP cleavage was analyzed as an indication of apoptosis in both EGFP and Kpnβ1-EGFP cells 48 h after cisplatin treatment. GAPDH was used as a control for loading. **D** Densitometric quantification of C-PARP relative to GAPDH from two independent experiments. **E** HeLa EGFP and Kpnβ1-EGFP cells were treated with cisplatin for 24 h, followed by western blot analysis to determine the levels of γH2AX, p53, p21 and Mcl-1. GAPDH was used as a control for loading. **F** Nuclear and cytoplasmic proteins were isolated from HeLa EGFP and Kpnβ1-EGFP cells and p53 and p21 levels determined by western blot analysis. TBP and GAPDH were used to control for even nuclear and cytoplasmic protein loading, respectively. **G** HeLa EGFP (a) and Kpnβ1-EGFP (b) cells were co-treated with Cisplatin and the p53 inhibitor Pifithrin α, and cell proliferation monitored 24 h later using the MTT assay. **H** HeLa EGFP (a) and Kpnβ1-EGFP (b) cells were transfected with control (ctl) or p21 siRNA, and 48 h later treated with Cisplatin for 24 h, whereafter cell proliferation was monitored using the MTT assay. Results shown represent the mean ± SEM of experiments (*p < 0.05)

Due to the fact that Kpnβ1 is involved in the nuclear import of proteins involved in apoptosis induced by chemotherapy, such as p53 [9, 28, 29], and since overexpression of Kpnβ1 alters the proliferation and cell cycle progression of cervical cancer cells, we next investigated whether Kpnβ1 overexpression may influence the effectiveness of cisplatin, an anticancer chemotherapy commonly used to treat patients with cervical cancer. To investigate whether upregulation of Kpnβ1 could alter the response of cancer cells to cisplatin treatment, cisplatin IC_50_ values were compared in HeLa EGFP and Kpnβ1-EGFP cells. The IC_50_ value for cisplatin was revealed to be 24.9 μM in EGFP cells and 13 μM in Kpnβ1-EGFP cells, respectively, suggesting that cells overexpressing Kpnβ1 have increased sensitivity to cisplatin (Fig. 5A, Additional file 2: Figure S2). Examination of cell viability confirmed these results; a significant decrease in cell viability at various concentrations of cisplatin was observed in cells expressing Kpnβ1-EGFP compared to those expressing EGFP (Fig. 5B). To determine whether the increased sensitivity to cisplatin treatment observed in cells overexpressing Kpnβ1 was associated with increased apoptosis, PARP cleavage after cisplatin treatment in EGFP and Kpnβ1-EGFP cells was analysed. Western blot analysis revealed enhanced PARP cleavage in cells overexpressing Kpnβ1 (Kpnβ1-EGFP) compared to cells expressing EGFP (Fig. 5C), which was confirmed by densitometric analysis (Fig. 5D). The effect of Kpnβ1 overexpression on the expression of various proteins that are known to be involved in the cisplatin response was next explored, including p53, p21 and Mcl-1. Examination of p53, p21 and Mcl-1 expression showed that cisplatin treatment lead to a concentration-dependent increase in p53 levels, and a concentration-dependent decrease in p21 and Mcl-1 levels, evident in both EGFP and Kpnβ1-EGFP cells but more prominent in the latter (Fig. 5E). Furthermore, Kpnβ1-EGFP cells showed higher p53 levels and lower p21 and Mcl-1 levels without cisplatin treatment (0 μM). As Kpnβ1 overexpression has been reported to cause mitotic abnormalities and induce genomic instability [7, 8], we investigated the DNA damage marker γH2AX after cisplatin treatment in Kpnβ1-EGFP compared to EGFP cells. Western blot analysis revealed that γH2AX levels were enhanced upon treatment of Kpnβ1-EGFP cells with cisplatin, indicative of higher levels of DNA damage in cells overexpressing Kpnβ1 (Fig. 5E).

The upregulation of p53 and downregulation of p21 in the Kpnβ1-EGFP-overexpressing cells, and in response to Cisplatin, warranted further investigation as p21 levels typically follow that of p53. To further explore the role of these proteins in the cellular response to Cisplatin, their localisation was examined. While p53 was found to be predominately nuclear in the EGFP and Kpnβ1-EGFP cells, p21 displayed more cytoplasmic localisation (Fig. 5F). p53 and p21 were next inhibited, using Pifithrin α and p21 siRNA respectively, and the effect on Cisplatin responsiveness monitored by MTT. Interestingly, p53 inhibition resulted in decreased cell death and p21 inhibition resulted in increased cell death in response to Cisplatin (Fig. 5G, H). Similar results were obtained using the CaSki EGFP and Kpnβ1-EGFP cells (Additional file 3: Figure S3). These results suggest that p53 acts to promote and p21 protect cells from Cisplatin toxicity. The increased sensitivity of Kpnβ1-EGFP-overexpressing cells to Cisplatin can thus, in part, be explained by the increased p53 and decreased p21 levels in these cells.

## Discussion

Changes in gene expression patterns are a significant driver of cancer progression. Thus, the identification of genes and pathways involved in and/or altered in this process will not only improve our understanding of cancer initiation and development, but will also help in the identification of novel anticancer drug targets. Previous studies have revealed Karyopherin β1 (Kpnβ1), the primary nuclear import protein, to be significantly overexpressed in a variety of cancer types including cervical [10], gastric [30], and breast cancers [13]. Furthermore, these studies reveal that inhibition of Kpnβ1 expression leads to cancer cell death, while inhibition in non-cancer cells has only a minor effect on cell viability [10]. These results suggest that Kpnβ1 has potential as an anticancer therapeutic target, thus warranting further research into the association between Kpnβ1 expression and cancer progression.

To better understand the role that Kpnβ1 plays in malignant transformation, we investigated the effects of Kpnβ1 overexpression on various biological processes associated with cellular transformation. A stable Kpnβ1 overexpressing cell line was generated and shown to result in enhanced nuclear activity of known Kpnβ1 cargoes. Surprisingly, this did not translate into an enhanced cancer phenotype as anticipated, but rather suppressed cancer cell proliferation, delayed cell cycle progression, and altered cell morphology and actin reorganization, resulting in increased cell adhesion. This suggests that while overexpression of Kpnβ1 is required for the cancer phenotype, further overexpression suppresses the malignancy of cervical cancer cells. This could be due to a number of reasons; such as an imbalance in nuclear import and export, resulting in altered nuclear localisation of cargo proteins whose localisation is critical for cell functioning. Alternatively, upregulation of Kpnβ1 could result in increased nuclear import of tumour suppressor proteins; indeed, it has been found that Kpnβ1 overexpression resulted in an increase in the nuclear activity of p53, a known Kpnβ1 cargo protein [21, 22]. Overexpression of Kpnβ1 may also result in the increased nuclear import of various other cell cycle proteins that are Kpnβ1 cargoes, such as cyclin B1 and cyclin E [31], whose altered localisation could greatly impact cell cycle progression. Interestingly, Kpnβ1 overexpression did not result in an increase in cell death in these cells, therefore the effects seen were due a reduction in rate of proliferation rather than an induction of cell death. Ultimately, the nuclear transport process is a tightly regulated process that is critical for cell functioning. Altering this process, by increasing the expression of Kpnβ1 alone, has negative cellular consequences for cervical cancer cells.

Normal cells overexpressing Kpnβ1 also displayed reduced cell proliferation, suggesting that Kpnβ1 alone is insufficient to promote cancer behaviour. In contrast to our results, Kodama et al. (2017) showed that stable overexpression of Kpnβ1 in ovarian cancer cells resulted in a significant increase in cell proliferation, and these authors suggest that Kpnβ1 acts as an oncogene [32]. It is possible that the effects observed due to Kpnβ1 overexpression may be specific to different cell types, and are likely subject to the levels of endogenous Kpnβ1 present in certain cell lines. Furthermore, while we show that the overexpressed Kpnβ1 in our study is functional, in that it results in the increased nuclear activity of Kpnβ1 cargoes, it is possible that the EGFP tag, fused to its C-terminus, may alter Kpnβ1 function or its ability to bind interaction partners, compared to the tag-less Kpnβ1 overexpressed by Kodama et al. (2017), hence contribute to the discrepancies between studies.

While Kpnβ1 overexpression alone appears to be insufficient to induce any of the biological phenotypes associated with cellular transformation, overexpression of additional members of the nuclear transport machinery may be required for cancer-associated changes. It is possible that in terms of nuclear import, cancer cells have already reached their maximum capability and thus further enhancement of various factors such as Kpnβ1, without strict control and regulation of all factors involved in nuclear transport (i.e. a precise balance), results in further cellular dysfunction.

A wide range of cancer processes have been shown to rely on Kpnβ1-mediated nuclear import. For example, a recent review shows how the Karyopherin proteins are involved in controlling a large number of epithelial-mesenchymal transition (EMT) promoting pathways [33]. Kpnβ1 has been shown to be involved in the nuclear translocation of a number of EMT-promoting proteins including Snail [34], Smad [35], and Notch [36], all of which play a role in increasing cancer cell invasion and metastasis. In addition to this, Kpnβ1 has also been shown to play a role in actin cytoskeleton regulation, by regulating the interaction between an actin binding kinesin (NabKin) and F-actin, demonstrating that Kpnβ1 likely plays a role in influencing cytoskeletal elements and structure [6, 37]. We showed that Kpnβ1 overexpression resulted in changes in cell morphology and actin reorganisation, with cells becoming smaller and more epithelial-like. In addition, Kpnβ1 overexpressing cells showed enhanced adhesion, as well as an increase in E-cadherin and decrease in Vimentin protein expression. These results suggest that overexpression of Kpnβ1 results in a mesenchymal-to-epithelial transition (MET), where cells transition from motile, more spindle-shaped mesenchymal cells to adherent, more compact/columnar epithelial cells. There is much evidence linking the EMT process with tumour progression and malignant transformation, as transitioning to a more mesenchymal state promotes cell invasion and metastasis (or cancer cell dissemination) [38]. More recently, evidence has started to emerge supporting the concept that MET may be important for metastatic colonization, but research remains limited. Thus, it appears that overexpression of Kpnβ1, beyond that which is already elevated, has negative consequences for the proliferation, progression and metastatic potential of cancer cells.

To identify whether overexpression of Kpnβ1 resulted in any changes to the cancer cell response to cisplatin treatment, HeLa EGFP and Kpnβ1-EGFP cells were treated with cisplatin and the effects on various processes observed, including cell viability and apoptosis. Our results revealed that Kpnβ1 overexpressing HeLa cells showed significantly reduced viability after treatment with cisplatin, and were more prone to cisplatin-induced apoptosis when compared to HeLa EGFP cells. This could be due to enhanced nuclear import of cargo proteins required for the apoptotic process, for example p53 [9, 28, 29]. p53 is involved in the activation of apoptosis induced by DNA-damaging agents, such as cisplatin [39]. We showed that HeLa cells overexpressing Kpnβ1 showed higher levels of p53 when treated with cisplatin, compared to cells expressing EGFP, and that the same trend in expression was seen with the DNA damage marker, γH2AX. Examination of the expression levels of anti-apoptotic proteins p21 and Mcl-1, revealed that both proteins were reduced in response to cisplatin treatment, and the reduction was more prominent in cells overexpressing Kpnβ1, suggesting that these cells are more sensitive to apoptotic signals. We also showed that stable overexpression of Kpnβ1 in synchronized HeLa cells resulted in a decrease in (or degradation of) Mcl-1 expression. Interestingly, a number of recent studies have shown that inhibition of Mcl-1 expression sensitizes cancer cells to cisplatin-induced apoptosis [40, 41]. We show that p21 inhibition sensitises cervical cancer cells to cisplatin treatment, which is in agreement with other studies showing that knockdown or inhibition of p21 expression significantly increases apoptosis of ovarian cancer and testicular cancer cells induced by cisplatin [42, 43]. On the other hand, other studies have reported p21 overexpression to increase Cisplatin sensitivity in lung adenocarcinoma cells [44, 45] and osteosarcoma cells [46], thus it is clear that different cancer cells use different cellular mechanisms to govern Cisplatin responsiveness. We also show that despite the HPV positive nature of HeLa and CaSki cells (and thus low p53 activity), p53 inhibition reduces cervical cancer cell sensitivity to Cisplatin, suggesting a role for p53 in mediating Cisplatin-induced cell death. This is corroborated by other studies, which show a role for p53 in Cisplatin-induced apoptosis [47]. However, p53, like p21, can exert anti-apoptotic effects in response to Cisplatin in other contexts [48]. Taken together, in our study, p53 and p21 enhance and protect cervical cancer cells from Cisplatin-induced cell death, respectively, thus the higher p53 and lower p21 in the Kpnβ1-EGFP-overexpressing cells likely contributes, at least in part, to the increased sensitivity of these cells to Cisplatin. Interestingly, Kodama et al. (2017) recently showed that inhibition of Kpnβ1 expression, in combination with paclitaxel treatment, synergistically reduced cell proliferation/viability in ovarian cancer cells [32].

Whilst we have previously shown that Kpnβ1 inhibition, genetically or pharmacologically, resulted in an induction of cervical cancer cell death and the appearance of mitotic defects [9, 10, 25], others have reported that exogenously expressed Kpnβ1 induced mitotic abnormalities, resulting in genomic instability [7, 8]. We report that Kpnβ1 overexpression results in increased levels of pSer10 Histone H3 in asynchronous cells, and delays cell cycle progression into G2/M in cells released from a thymidine block, suggesting that mitotic processes are somewhat impaired in these cells. However, further overexpression of Kpnβ1-EGFP, beyond that achieved in this study, resulted in distinct mitotic defects and cell death (data not shown), further reinforcing that Kpnβ1 overexpression is detrimental to cervical cancer cell biology. It is likely that cancer cells require a precise balance of Kpnβ1 expression, and that perturbation of this balance in either direction has deleterious effects on cancer cell survival.

## Conclusion

To our knowledge this is the first study to show that overexpression of Kpnβ1 provides no growth advantage to cervical cancer cells or non-cancer epithelial cells. In fact, when Kpnβ1 is overexpressed in cervical cancer cells, negative effects associated with a variety of biological processes are observed, including reduced cell proliferation, delayed cell cycle progression, altered cellular morphology, and increased cellular adhesion. Furthermore, Kpnβ1 overexpression enhances cervical cancer cell sensitivity to cisplatin, mediated via increased p53 and decreased p21 protein levels. Ultimately, it appears as if a tight regulation of Kpnβ1 is essential for the correct functioning of cancer cells and for the responsiveness of these cells to chemotherapeutic agents, like Cisplatin.

## Additional files

Additional file 1: **Figure S1**. Overexpression of Kpnβ1 results in changes in the morphology and adhesion properties of CaSki cervical cancer cells. **A:** Phase contrast images showing CaSki EGFP and Kpnβ1-EGFP cells, taken 48 h post plating. Cells were viewed at 20 x magnification using the Zeiss Primovert inverted phase microscope. **B**: Quantification of relative CaSki cell area ± SEM of forty cells from each condition was performed using the AxioVision 4.7 software (*p < 0.05). **C:** Fluorescent staining of polymeric F-actin using phalloidin (red) in EGFP and Kpnβ1-EGFP expressing CaSki cells. DAPI stain was used to visualize the cell nuclei (blue). **D:** Quantification of the number of cytoplasmic protrusions from the captured fluorescent images. Results shown represent the mean ± SEM over fifteen fields of view (*p < 0.05). **E**: Relative cell adhesion in CaSki EGFP and Kpnβ1-EGFP cells. Adherent cells were fixed (after removing non-adherent cells by washing) and stained with 0.5% crystal violet solution. Cells over ten fields of view, viewed at 10 x magnification, were counted using ImageJ and normalized to unwashed cells. Results shown represent the mean ± SEM (*p < 0.05)**. F:** Western blot analysis was used to determine the expression levels of E-cadherin and Vimentin in CaSki Kpnβ1-overexpressing cells. GAPDH was used as a control for loading. **G:** An in vitro scratch wound healing assay was performed and showed no change in migration of CaSki EGFP and Kpnβ1-EGFP cells within a 24 h period. **H:** Quantification of the scratch wound healing assay in G. (PPTX 627 kb)

Additional file 2: **Figure S2**. Overexpression of Kpnβ1 results in increased sensitivity to Cisplatin. Dose-response curves are shown after treatment of HeLa EGFP- and HeLa Kpnβ1-EGF-expressing cells with Cisplatin. (PPTX 42 kb)

Additional file 3: **Figure S3**. p53 and p21 inhibition reduces and enhances CaSki EGFP and Kpnβ1-EGFP cell sensitivity to Cisplatin, respectively. **A:** CaSki EGFP (a) and Kpnβ1-EGFP (b) cells were co-treated with Cisplatin and the p53 inhibitor Pifithrin α, and cell proliferation monitored 24 h later using the MTT assay. **B:** CaSki EGFP (a) and Kpnβ1-EGFP (b) cells were transfected with control (ctl) or p21 siRNA, and 48 h later treated with Cisplatin for 24 h, whereafter cell proliferation was monitored using the MTT assay. Results shown represent the mean ± SEM of experiments (*p < 0.05). (PPTX 59 kb)

## Abbreviations

ATCC: American Type Culture Collection
CANSA: Cancer Association of South Africa
cNLS: Classical NLS
Ctl: Control
DMEM: Dulbecco’s Modified Eagle’s Medium
EMT: Epithelial-to-mesenchymal transition
F-actin: Filamentous actin
FCS: Fetal Calf Serum
IRES: Internal ribosome entry site
Kpnα: Karyopherin α
Kpnβ1: Karyopherin β1
MET: Mesenchymal-to-epithelial transition
NLS: Nuclear localisation sequence
NPC: Nuclear pore complex
Nups: Nucleoporins
pHisH3: PS10-Histone-H3

## References

[1] Mosammaparast N, Pemberton LF (2004). Karyopherins: from nuclear-transport mediators to nuclear-function regulators. Trends Cell Biol.

[2] Görlich D, Henklein P, Laskey RA, Hartmann E (1996). A 41 amino acid motif in importin-alpha confers binding to importin-beta and hence transit into the nucleus. EMBO J.

[3] Freitas N, Cunha C (2009). Mechanisms and signals for the nuclear import of proteins. Curr Genomics.

[4] Moroianu J, Blobel G, Radu A (1996). Nuclear protein import: ran-GTP dissociates the karyopherin alphabeta heterodimer by displacing alpha from an overlapping binding site on beta. Proc Natl Acad Sci U S A.

[5] Harel A, Forbes DJ (2004). Importin beta: conducting a much larger cellular symphony. Mol Cell.

[6] Forbes DJ, Travesa A, Nord MS, Bernis C (2015). Nuclear transport factors: global regulation of mitosis. Curr Opin Cell Biol.

[7] Ciciarello M, Mangiacasale R, Thibier C, Guarguaglini G, Marchetti E, Di Fiore B (2004). Importin beta is transported to spindle poles during mitosis and regulates ran-dependent spindle assembly factors in mammalian cells. J Cell Sci.

[8] Roscioli E, Di Francesco L, Bolognesi A, Giubettini M, Orlando S, Harel A (2012). Importin-β negatively regulates multiple aspects of mitosis including RANGAP1 recruitment to kinetochores. J Cell Biol.

[9] Angus L, van der Watt PJ, Leaner VD (2014). Inhibition of the nuclear transporter, Kpnβ1, results in prolonged mitotic arrest and activation of the intrinsic apoptotic pathway in cervical cancer cells. Carcinogenesis.

[10] van der Watt PJ, Maske CP, Hendricks DT, Parker MI, Denny L, Govender D (2009). The Karyopherin proteins, Crm1 and Karyopherin β1, are overexpressed in cervical cancer and are critical for cancer cell survival and proliferation. Int J Cancer.

[11] Kuusisto HV, Wagstaff KM, Alvisi G, Roth DM, Jans DA (2012). Global enhancement of nuclear localization-dependent nuclear transport in transformed cells. FASEB J.

[12] Smith ER, Cai KQ, Smedberg JL, Ribeiro MM, Rula ME, Slater C (2010). Nuclear entry of activated MAPK is restricted in primary ovarian and mammary epithelial cells. PLoS One.

[13] Kuusisto HV, Jans DA (1853). Hyper-dependence of breast cancer cell types on the nuclear transporter importin β1. Biochim Biophys Acta.

[14] Hobbs S, Jitrapakdee S, Wallace JC (1998). Development of a Bicistronic vector driven by the human polypeptide chain elongation factor 1α promoter for creation of stable mammalian cell lines that express very high levels of recombinant proteins. Biochem Biophys Res Commun.

[15] Beals CR, Clipstone NA, Ho SN, Crabtree GR (1997). Nuclear localization of NF-ATc by a calcineurin-dependent, cyclosporin-sensitive intramolecular interaction. Genes Dev.

[16] Ichida M, Finkel T (2001). Ras regulates NFAT3 activity in cardiac myocytes. J Biol Chem.

[17] Maritz MF, van der Watt PJ, Holderness N, Birrer MJ, Leaner VD (2011). Inhibition of AP-1 suppresses cervical cancer cell proliferation and is associated with p21 expression. Biol Chem.

[18] el-Deiry WS, Tokino T, Velculescu VE, Levy DB, Parsons R, Trent JM (1993). WAF1, a potential mediator of p53 tumor suppression. Cell.

[19] Forwood JK, Lam MHC, Jans DA (2001). Nuclear import of Creb and AP-1 transcription factors requires importin-β1 and ran but is independent of importin-α. Biochemistry.

[20] Liang P, Zhang H, Wang G, Li S, Cong S, Luo Y (2013). KPNB1, XPO7 and IPO8 mediate the translocation of NF-κB/p65 into the nucleus. Traffic.

[21] Liang SH, Clarke MF (1999). A bipartite nuclear localization signal is required for p53 nuclear import regulated by a carboxyl-terminal domain. J Biol Chem.

[22] Kim I, Kim D, Han S, Chin M, Nam H, Cho H (2000). Truncated form of importin α identified in breast Cancer cell inhibits nuclear import of p53. J Biol Chem.

[23] Harley ME, Allan LA, Sanderson HS, Clarke PR (2010). Phosphorylation of mcl-1 by CDK1–cyclin B1 initiates its Cdc20-dependent destruction during mitotic arrest. EMBO J.

[24] Nijhawan D, Fang M, Traer E, Zhong Q, Gao W, Du F (2003). Elimination of mcl-1 is required for the initiation of apoptosis following ultraviolet irradiation. Genes Dev.

[25] van der Watt PJ, Chi A, Stelma T, Stowell C, Strydom E, Carden S (2016). Targeting the nuclear import receptor Kpnβ1 as an anticancer therapeutic. Mol Cancer Ther.

[26] van Roy F, Berx G (2008). The cell-cell adhesion molecule E-cadherin. Cell Mol Life Sci.

[27] Kidd ME, Shumaker DK, Ridge KM (2014). The role of vimentin intermediate filaments in the progression of lung Cancer. Am J Respir Cell Mol Biol.

[28] Johnstone RW, Ruefli AA, Lowe SW (2002). Apoptosis: a link between cancer genetics and chemotherapy. Cell.

[29] Seitz SJ, Schleithoff ES, Koch A, Schuster A, Teufel A, Staib F (2010). Chemotherapy-induced apoptosis in hepatocellular carcinoma involves the p53 family and is mediated via the extrinsic and the intrinsic pathway. Int J Cancer.

[30] Zhu J, Wang Y, Huang H, Yang Q, Cai J, Wang Q (2016). Upregulation of KPNβ1 in gastric cancer cell promotes tumor cell proliferation and predicts poor prognosis. Tumor Biol.

[31] Moore JD, Yang J, Truant R, Kornbluth S (1999). Nuclear import of Cdk/cyclin complexes: identification of distinct mechanisms for import of Cdk2/cyclin E and Cdc2/cyclin B1. J Cell Biol.

[32] Kodama M, Kodama T, Newberg JY, Katayama H, Kobayashi M, Hanash SM (2017). In vivo loss-of-function screens identify KPNB1 as a new druggable oncogene in epithelial ovarian cancer. Proc Natl Acad Sci.

[33] Azmi AS (2013). Unveiling the role of nuclear transport in epithelial-to-mesenchymal transition. Curr Cancer Drug Targets.

[34] Yamasaki H, Sekimoto T, Ohkubo T, Douchi T, Nagata Y, Ozawa M (2005). Zinc finger domain of snail functions as a nuclear localization signal for importin β-mediated nuclear import pathway. Genes Cells.

[35] Hill CS (2009). Nucleocytoplasmic shuttling of Smad proteins. Cell Res.

[36] Huenniger K, Krämer A, Soom M, Chang I, Köhler M, Depping R (2010). Notch1 signaling is mediated by importins alpha 3, 4, and 7. Cell Mol Life Sci.

[37] Samwer M, Dehne H-J, Spira F, Kollmar M, Gerlich DW, Urlaub H (2013). The nuclear F-actin interactome of Xenopus oocytes reveals an actin-bundling kinesin that is essential for meiotic cytokinesis. EMBO J.

[38] Larue L, Bellacosa A (2005). Epithelial–mesenchymal transition in development and cancer: role of phosphatidylinositol 3′ kinase/AKT pathways. Oncogene.

[39] Han JY, Chung YJ, Park SW, Kim JS, Rhyu MG, Kim HK (1999). The relationship between cisplatin-induced apoptosis and p53, bcl-2 and bax expression in human lung cancer cells. Korean J Intern Med.

[40] Zhang F, Shen M, Yang L, Yang X, Tsai Y, Keng PC (2017). Simultaneous targeting of ATM and mcl-1 increases cisplatin sensitivity of cisplatin-resistant non-small cell lung cancer. Cancer Biol Ther.

[41] Yu X, Li W, Xia Z, Xie L, Ma X, Liang Q (2017). Targeting MCL-1 sensitizes human esophageal squamous cell carcinoma cells to cisplatin-induced apoptosis. BMC Cancer.

[42] Xia X, Ma Q, Li X, Ji T, Chen P, Xu H (2011). Cytoplasmic p21 is a potential predictor for cisplatin sensitivity in ovarian cancer. BMC Cancer.

[43] Koster R, di Pietro A, Timmer-Bosscha H, Gibcus JH, van den Berg A, Suurmeijer AJ (2010). Cytoplasmic p21 expression levels determine cisplatin resistance in human testicular cancer. J Clin Invest.

[44] Liu Z, Sun M, Lu K, Liu J, Zhang M, Wu W (2013). The long noncoding RNA HOTAIR contributes to cisplatin resistance of human lung adenocarcinoma cells via downregualtion of p21WAF1/CIP1 expression. PLoS One.

[45] Wang H, Zhu LJ, Yang YC, Wang ZX, Wang R (2014). MiR-224 promotes the chemoresistance of human lung adenocarcinoma cells to cisplatin via regulating G1/S transition and apoptosis by targeting p21 WAF1/CIP1. Br J Cancer.

[46] Ding Y, Wang Y, Chen J, Hu Y, Cao Z, Ren P (2014). P21 overexpression sensitizes osteosarcoma U2OS cells to cisplatin via evoking caspase-3 and Bax/Bcl-2 cascade. Tumor Biol.

[47] Wesierska-Gadek J, Schloffer D, Kotala V, Horky M (2002). Escape of p53 protein from E6-mediated degradation in HeLa cells after cisplatin therapy. Int J Cancer.

[48] Di Pietro A, Koster R, Boersma-van Eck W, Dam WA, Mulder NH, Gietema JA (2012). Pro- and anti-apoptotic effects of p53 in cisplatin-treated human testicular cancer are cell context-dependent. Cell Cycle.

