# Supplementary material for: A tight balance of Karyopherin β1 expression is required in cervical cancer cells
Source: BMC Cancer. 2018 Nov 16;18:1123. doi: 10.1186/s12885-018-5044-8 (PMC6240311; doi:10.1186/s12885-018-5044-8)
Supplement: Supplementary file 1 — Figure S1. Overexpression of Kpnβ1 results in changes in the morphology and adhesion properties of CaSki cervical cancer cells. A: Phase contrast images showing CaSki EGFP and Kpnβ1-EGFP cells, taken 48 h post plating. Cells were viewed at 20 x magnification using the Zeiss Primovert inverted phase microscope. B: Quantification of relative CaSki cell area ± SEM of forty cells from each condition was performed using the AxioVision 4.7 software (*p < 0.05). C: Fluorescent staining of polymeric F-actin using phalloidin (red) in EGFP and Kpnβ1-EGFP expressing CaSki cells. DAPI stain was used to visualize the cell nuclei (blue). D: Quantification of the number of cytoplasmic protrusions from the captured fluorescent images. Results shown represent the mean ± SEM over fifteen fields of view (*p < 0.05). E: Relative cell adhesion in CaSki EGFP and Kpnβ1-EGFP cells. Adherent cells were fixed (after removing non-adherent cells by washing) and stained with 0.5% crystal violet solution. Cells over ten fields of view, viewed at 10 x magnification, were counted using ImageJ and normalized to unwashed cells. Results shown represent the mean ± SEM (*p < 0.05). F: Western blot analysis was used to determine the expression levels of E-cadherin and Vimentin in CaSki Kpnβ1-overexpressing cells. GAPDH was used as a control for loading. G: An in vitro scratch wound healing assay was performed and showed no change in migration of CaSki EGFP and Kpnβ1-EGFP cells within a 24 h period. H: Quantification of the scratch wound healing assay in G. (PPTX 627 kb) [file 12885_2018_5044_MOESM1_ESM.pptx]

## Slide 1
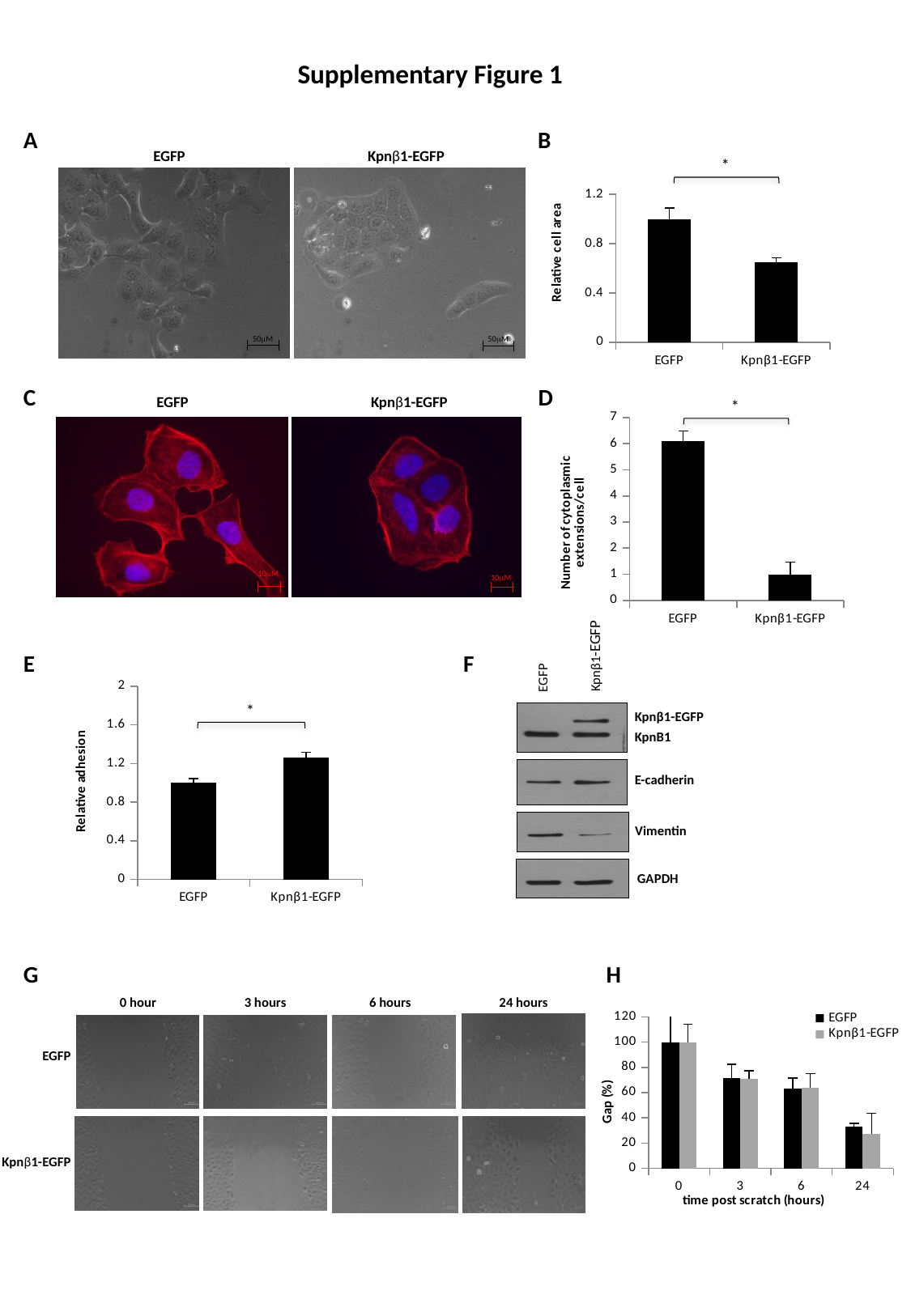

Supplementary Figure 1
A
B
EGFP
Kpnβ1-EGFP
### Chart
| Category | |
|---|---|
| EGFP | 1.0 |
| Kpnβ1-EGFP | 0.650601764891607 |*
50μM
50μM
D
C
EGFP
Kpnβ1-EGFP
*
### Chart
| Category | |
|---|---|
| EGFP | 6.1 |
| Kpnβ1-EGFP | 1.0 |
10μM
10μM
Kpnβ1-EGFP
EGFP
Kpnβ1-EGFP
KpnB1
E-cadherin
Vimentin
GAPDH
E
F
### Chart
| Category | |
|---|---|
| EGFP | 1.0 |
| Kpnβ1-EGFP | 1.264197530864197 |*
G
H
0 hour
3 hours
6 hours
24 hours
EGFP
Kpnβ1-EGFP
### Chart
| Category | | |
|---|---|---|
| 0 | 100.0 | 100.0 |
| 3 | 71.37276197988193 | 70.88174414184807 |
| 6 | 63.24041711280851 | 63.78671569548927 |
| 24 | 33.27615311533621 | 27.33610122884827 |
