# Supplementary figures and images for: A tight balance of Karyopherin β1 expression is required in cervical cancer cells
Source: BMC Cancer. 2018 Nov 16;18:1123. doi: 10.1186/s12885-018-5044-8 (PMC6240311; doi:10.1186/s12885-018-5044-8)

## Slide 1
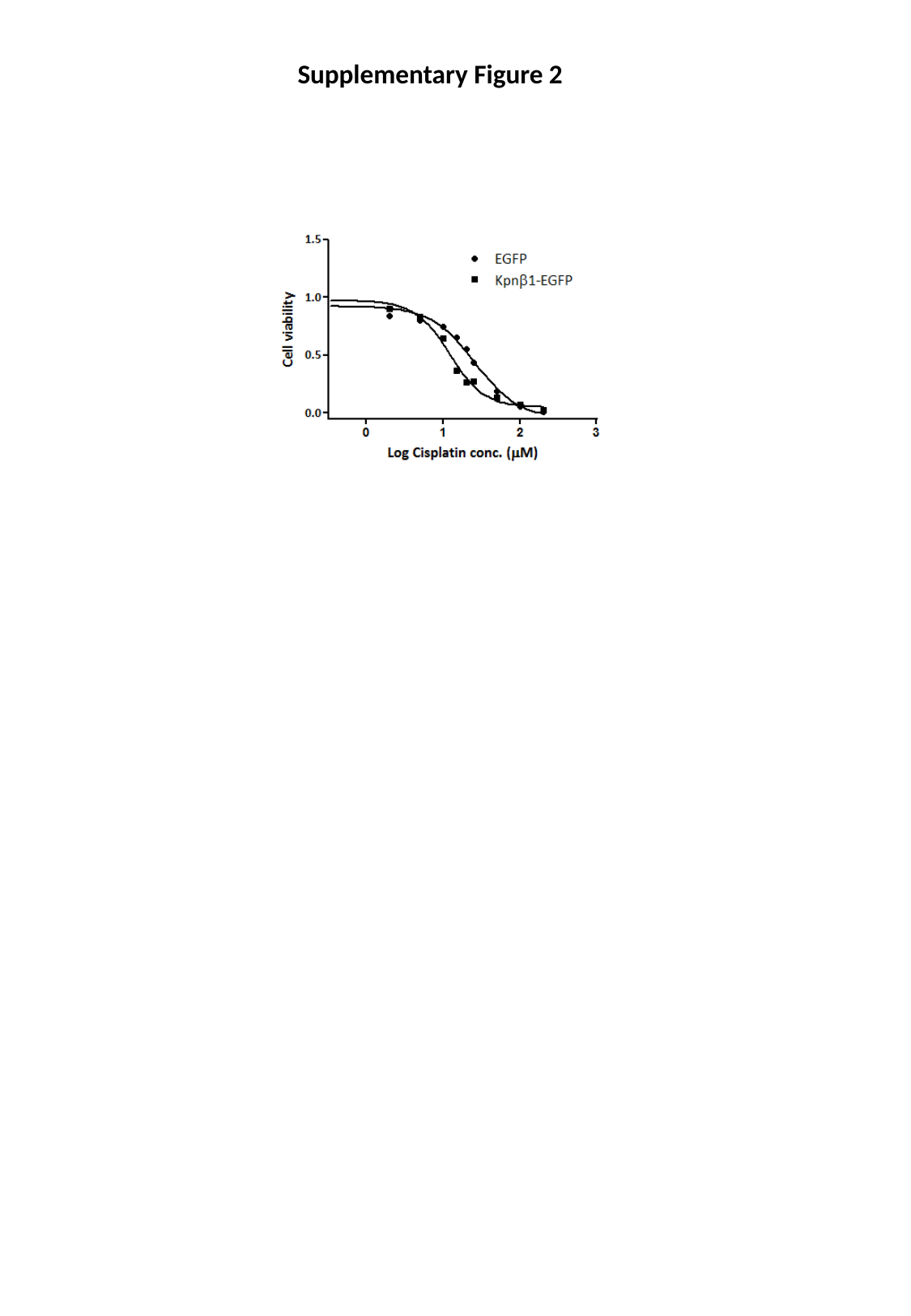

Supplementary Figure 2

Supplement: Supplementary file 2 — Figure S2. Overexpression of Kpnβ1 results in increased sensitivity to Cisplatin. Dose-response curves are shown after treatment of HeLa EGFP- and HeLa Kpnβ1-EGF-expressing cells with Cisplatin. (PPTX 42 kb) [file 12885_2018_5044_MOESM2_ESM.pptx]
