# Supplementary material for: A tight balance of Karyopherin β1 expression is required in cervical cancer cells
Source: BMC Cancer. 2018 Nov 16;18:1123. doi: 10.1186/s12885-018-5044-8 (PMC6240311; doi:10.1186/s12885-018-5044-8)
Supplement: Supplementary file 3 — Figure S3. p53 and p21 inhibition reduces and enhances CaSki EGFP and Kpnβ1-EGFP cell sensitivity to Cisplatin, respectively. A: CaSki EGFP (a) and Kpnβ1-EGFP (b) cells were co-treated with Cisplatin and the p53 inhibitor Pifithrin α, and cell proliferation monitored 24 h later using the MTT assay. B: CaSki EGFP (a) and Kpnβ1-EGFP (b) cells were transfected with control (ctl) or p21 siRNA, and 48 h later treated with Cisplatin for 24 h, whereafter cell proliferation was monitored using the MTT assay. Results shown represent the mean ± SEM of experiments (*p < 0.05). (PPTX 59 kb) [file 12885_2018_5044_MOESM3_ESM.pptx]

## Slide 1
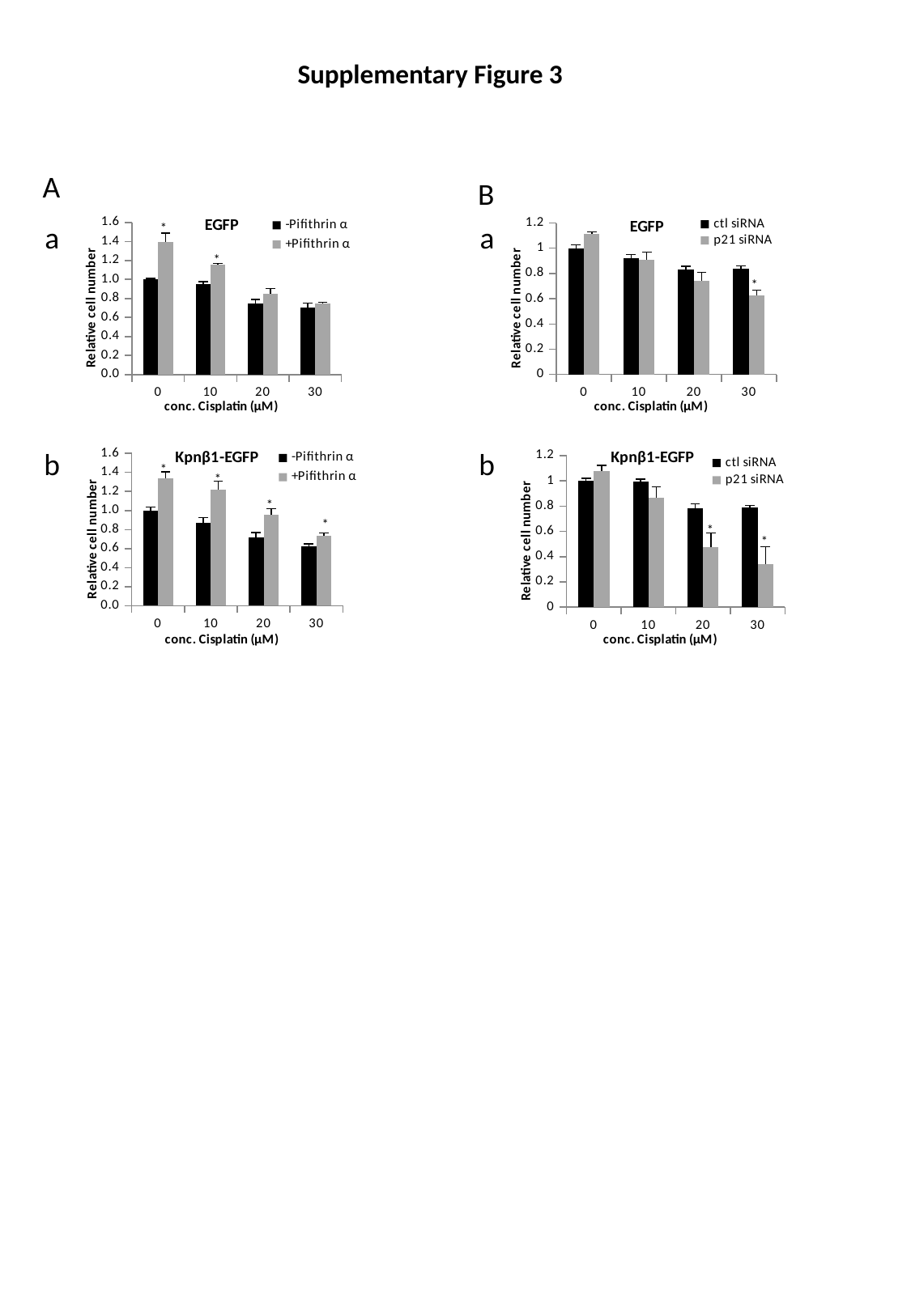

Supplementary Figure 3
A
B
a
### Chart: EGFP
| Category | | |
|---|---|---|
| 0 | 1.0019607843137255 | 1.3960784313725487 |
| 10 | 0.9529411764705882 | 1.1529411764705881 |
| 20 | 0.7509803921568627 | 0.8529411764705883 |
| 30 | 0.7039215686274509 | 0.7431372549019607 |a
### Chart: EGFP
| Category | | |
|---|---|---|
| 0 | 1.0000000000000004 | 1.1132427391420199 |
| 10 | 0.924060751398881 | 0.9100719424460435 |
| 20 | 0.8341326938449244 | 0.7428723687716494 |
| 30 | 0.8354649613642421 | 0.626298960831335 |*
*
*
b
b
### Chart: Kpnβ1-EGFP
| Category | | |
|---|---|---|
| 0 | 1.0 | 1.3392857142857142 |
| 10 | 0.8730158730158729 | 1.222222222222222 |
| 20 | 0.7202380952380951 | 0.9523809523809523 |
| 30 | 0.6249999999999999 | 0.7321428571428571 |
### Chart: Kpnβ1-EGFP
| Category | | |
|---|---|---|
| 0 | 1.0000000000000002 | 1.0809549945115258 |
| 10 | 0.9972557628979145 | 0.8655323819978047 |
| 20 | 0.781833150384193 | 0.4744785949506038 |
| 30 | 0.7873216245883643 | 0.3427552140504939 |*
*
*
*
*
*
